# A novel hepadnavirus in domestic dogs

**DOI:** 10.1038/s41598-022-06842-z

**Published:** 2022-02-21

**Authors:** Georgia Diakoudi, Paolo Capozza, Gianvito Lanave, Francesco Pellegrini, Barbara Di Martino, Gabriella Elia, Nicola Decaro, Michele Camero, Paola Ghergo, Fabio Stasi, Alessandra Cavalli, Maria Tempesta, Vanessa R. Barrs, Julia Beatty, Krisztián Bányai, Cristiana Catella, Maria S. Lucente, Alessio Buonavoglia, Giovanna Fusco, Vito Martella

**Affiliations:** 1 University of Bari, Valenzano, Italy; 2 University of Teramo, Teramo, Italy; 3 ACV Triggiano S.R.L., Triggiano, Italy; 4 Biotechlab, Brindisi, Italy; 5 Department of Veterinary Clinical Sciences, City University of Hong Kong, Kowloon Tong, , Hong Kong; 6 Veterinary Medical Research Institute, Budapest, Hungary; 7 University of Veterinary Medicine, Budapest, Hungary; 8 University of Bologna, Bologna, Italy; 9 Istituto Zooprofilattico Sperimentale del Mezzogiorno, Portici, Italy

**Keywords:** Viral infection, Diseases, Infectious diseases, Hepatitis, Viral hepatitis, Microbiology, Virology, Hepatitis B virus

## Abstract

Hepadnaviruses have been identified in several animal species. The hepadnavirus prototype, human hepatitis B virus (HBV), is a major public health problem associated with chronic liver diseases and hepatocellular carcinoma. Recently, a novel hepadnavirus, similar to HBV, was identified in domestic cats. Since several pathogens can be shared between cats and dogs, we hypothesized that dogs could also harbor hepadnaviruses and we tested a collection of canine sera with multiple molecular strategies. Overall, hepadnavirus DNA was identified in 6.3% (40/635) of canine serum samples, although the viral load in positive sera was low (geometric mean of 2.70 × 10^2^ genome copies per mL, range min 1.36 × 10^2^—max 4.03 × 10^4^ genome copies per mL). On genome sequencing, the canine hepadnaviruses revealed high nucleotide identity (about 98%) and similar organization to the domestic cat hepadnavirus. Altered hepatic markers were found in hepadnavirus-positive dogs, although the role of hepadnavirus in canine health remains to be elucidated.

## Introduction

Members of the genus *Orthohepadnavirus*, family *Hepadnaviridae*, are DNA viruses with a partially double-stranded circular genome, identified in several animal species. The prototype species, hepatitis B virus (HBV), is a major global health problem causing life-threatening acute and chronic liver infection in humans with increased risk of liver cirrhosis and hepatocellular carcinoma. The genome of HBV contains two large open reading frames (ORFs) encoding the surface (preS/S) protein and the polymerase (P), and two smaller ORFs encoding the precore/core (preC/C) and X proteins^1^.

In 2018, a novel hepadnavirus, similar to HBV, was identified in a domestic cat with lymphoma (domestic cat hepadnavirus, DCH) in Australia^2^. DCH infection seems to be common in cats with immunosuppression and hepatopathy^3,4^. Antibodies specific to HBV have been found in canine sera^5–8^ suggesting exposure of dogs to HBV or HBV-like viruses. Since canine and feline viromes may partially overlap, with some viruses circulating among canids and felids both in domestic and wild environments^9^, we hypothesized that HBV-like viruses might also be harbored by dogs.

## Results and discussion

Overall, a total of 40/635 (6.3%) sera tested positive in the quantitative (qPCR) screening and domestic dog hepadnavirus (DDH) DNA was found at low titer. The geometric mean value of DDH viremia in canine sera was 2.70 × 10^2^ copies per mL (range min 1.36 × 10^2^—max 4.03 × 10^4^ DNA copies per mL). Out of 40 DDH-infected dogs, we could retrieve information on hematologic and serum biochemical parameters for 23 animals (Supplementary Table 1). Eleven dogs (47.8%) had either increased alanine transaminase and/or aspartate transaminase and 14 (60.8%) had increased alkaline phosphatase (Table 1). Ninety-six % (38/40) of DDH-positive dogs were older than 1 year with 57% (23/40) being older than 7 years and 70% (28/40) were male.

**Table 1:** Biochemical profile of the DDH positive canine sera.

Sample ID	ALT	AST	ALP	GGT	Bil
455–10					
570–31					
570					
43–16			*	*	
43–18					
43			*		
232–5					
356–25		*			
356–27			*		
477–1	*	*	*		
477–4		*			
477–5	*		*		
477–8			*		
477–10	*		*		
477–30	*		*	*	
477–34		*	*	*	*
112–12	*				
112–20			*		
112–36	*	*	*	*	*
112–37	*	*	*	*	*
112–38	*		*		
112–39			*		
196–38					

Asterisk indicates values exceeding the normal upper limits for alanine aminotransferase (64 UI/L), aspartate aminotransferase (54 UI/L), alkaline phosphatase (7 UI/L), gammaglutamyl transpeptidase (7.0 UI/L) and total bilirubin (0.30 mg/dL).

*ALT* alanine transaminase; *AST* aspartate transaminase; *ALP* alkaline phosphatase; *GGT* gamma glutamyl trasnpeptidase; *Bil* Bilirubin.

**Figure 1:**
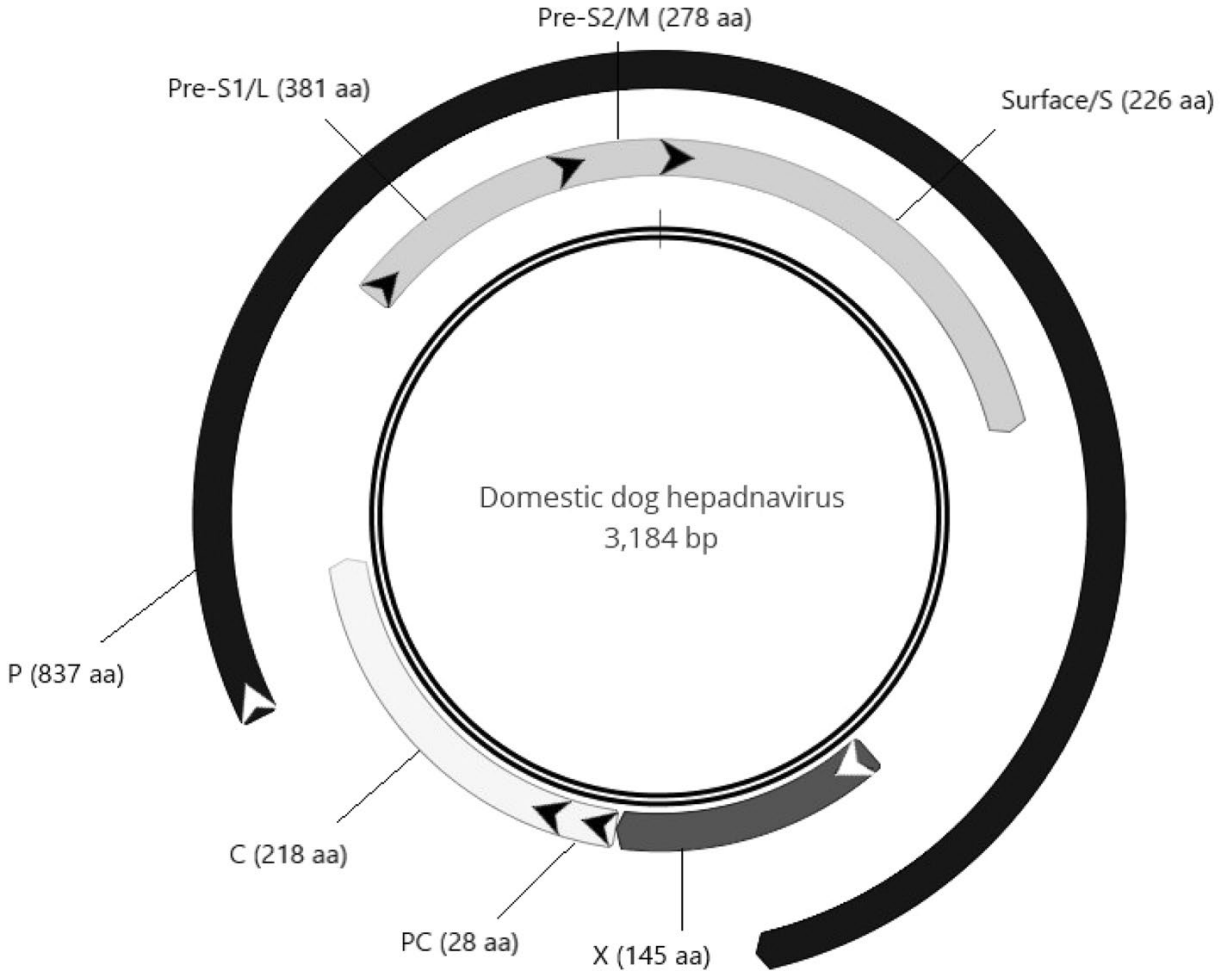
Genome organization of the DDH. The complete genome consists of 3184 bp. The proteins encoded by the polymerase (P), surface (S), core (C) and X ORFs are labelled in grey shades. The predicted Pre-S1/L (large), Pre-S2/M (middle) and Surface/S forms of the S protein are indicated. Also, the Pre-core (PC) region is shown. The length of each protein is indicated in amino acids (aa). The arrows indicate the position of the initiator codons and the ORF direction.

**Figure 2:**
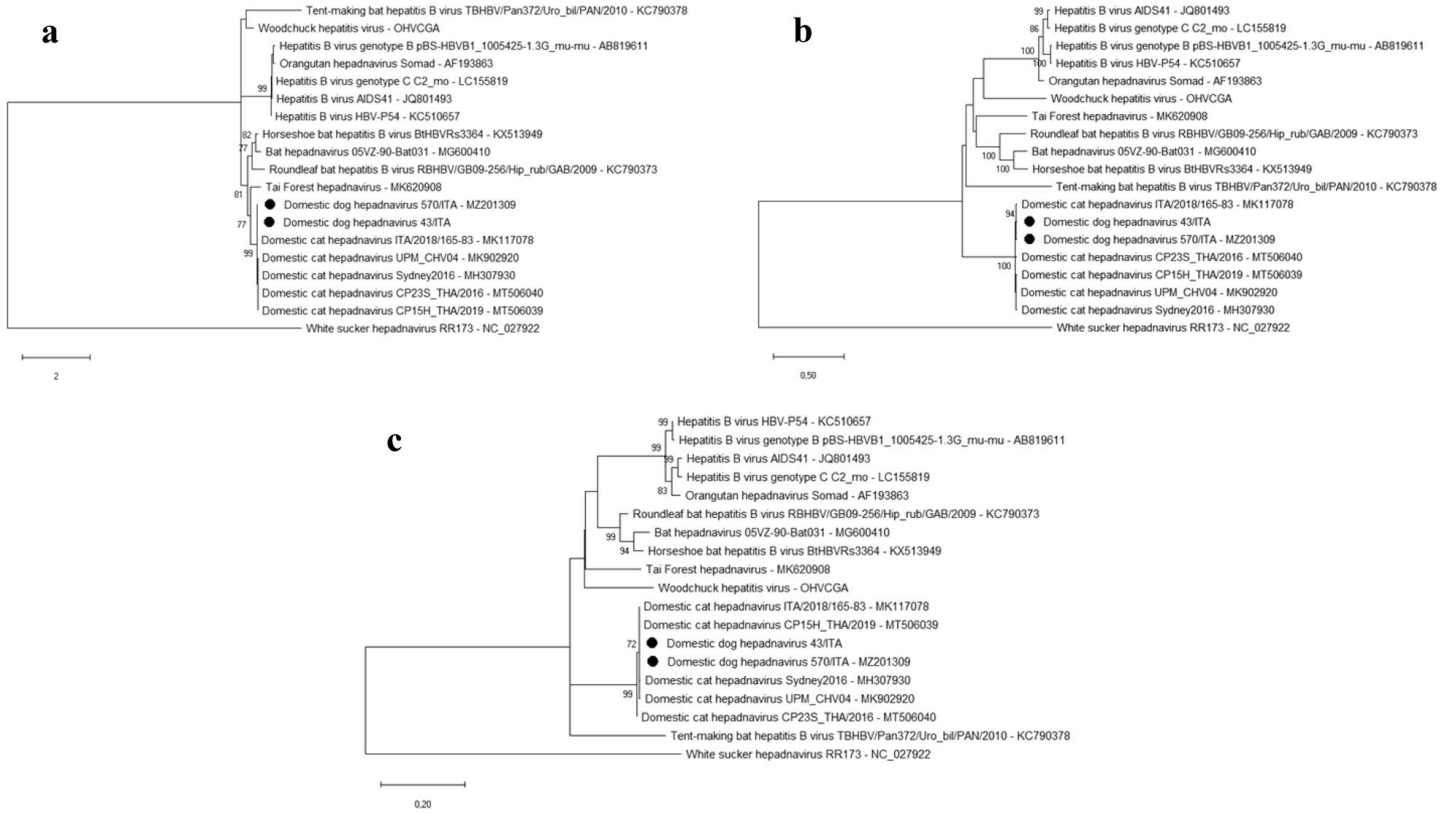
Phylogenetic trees based on three different gene targets, (**a**) core (partial-length), (**b**) polymerase (partial-length) and (**c**) surface (full-length), of hepadnaviruses retrieved from the GenBank database. GenBank accession numbers are provided for reference strains. The trees were generated using the maximum likelihood method, Hasegawa-Kishino-Yano model with a gamma distribution and invariant sites, and bootstrapping up to 1000 replicates. Bootstrap values > 70% are shown. Italian DDH strains 43/ITA and 570/ITA (GenBank accession no. MZ201309) are indicated by black bullets. White Sucker hepadnavirus (NC_027922) was used as outgroup. Scale bar indicates nt substitutions per site.

The complete genome of the DDH strain 570/ITA (GenBank accession no. MZ201309) was of 3184 bp in length (Fig. 1). The virus displayed 98.0% nucleotide (nt) identity at the full genome level to the Italian DCH strain ITA/2018/165-83 (GenBank accession no. MK117078) and 96.9% nt identity to the Australian DCH reference strain Sydney2016 (GenBank accession no. MH307930), respectively. For an additional DDH strain, 43/ITA, we were able to reconstruct a contig spanning about 80.7% (2572 nt) of the genome, including the partial C and P genes and the complete S gene. Sequence analysis showed that the DDH 43/ITA strain was 97.7%, 97.8% and 98.7% nt identical to the C, P and S gene sequences of the DDH 570/ITA strain, respectively. Upon phylogenetic analysis of the C, S and P ORFs, the two DDH strains were closely related to each other and intermingled with other DCH strains (Fig. 2). These findings would suggest the possibility of a free circulation of hepadnaviruses among domestic carnivores, rather than different viral species with a specific host range. A similar situation has been observed in equids, with similar hepadnaviruses circulating in zebras and donkeys^10^.

A limit of this study was the fact that we identified DDH only in the sera of dogs, whilst we did not test tissues or organs. Screening of liver biopsies from animals with hepatic diseases could be helpful to gather more precise information on this virus. However, when testing in Western blot (WB) a subset (*n* = 20) of the DDH-positive (viremic) sera, a total of 13/20 (65.0%) reacted with DCH core protein. Of these, 2 sera (15.4%, 2/13) were positive only for IgM, 3 samples (23.0%, 3/13) reacted only for IgG and an additional 8 sera (61.5%, 8/13) showed reactivity both for IgM or IgG.

Antibodies specific to HBV have previously been reported in dogs in two studies, dating back to the 1980s^5,6^. In a 1983 study from USA, a total of 172 sera from 33 animal species were screened and HBV S antigen (HBsAg) and antibodies to the C protein (anti-HBc) were not detected. However, 48% (82/172 serum samples from 19 animal species) contained antibodies against the S protein (anti-HBs), including 75% of the tested dog sera, collected from inbred Beagles from Maryland^5^. Similar findings were reported in a 1983 study from Taiwan. Anti-HBs for HBV were detected in 39/66 (59.1%) sera from stray dogs, with older animals being more prone to HBV infection^6^. The sera of the dogs tested negative for HBsAg and anti-HBc. Likewise, in a small sero-epidemiological survey in Iraq, 2020, 9% (7/78) of stray dogs had anti-HBs and serological positivity was correlated with increase in hepatic markers^8^.

In a 2019 study in Brazil, using an ELISA kit able to detect HBV markers (HBsAg and total anti-HBc), HBsAg was detected in 5.8% (11/189) of stray dogs and total anti-HBc was detected in 10% (19/189) of the animals. Using two PCR assays with primers targeted to the preS/S1 genomic region and to the core gene of HBV, hepadnavirus DNA was identified in 10% of dogs. On partial sequencing (about 1 kb) of the S gene, the virus was 98.2% similar at the nt level to HBV. Also, the DNA of similar HBV-like viruses was detected in sera of 5.9% (8/136) of pigs^7^. Notably, the sequence of the Brazilian hepadnavirus was distantly related (61.2% nt) to the sequences of the DDH strains identified in our investigation.

Interpreting these data is challenging, considering the diversity of sampling in terms of numbers of animals and inclusion criteria, and the different diagnostic strategies. In all the aforementioned studies, antigens and immunological reagents raised to HBV were used. Some authors hypothesized that the presence of anti-HBs in animal sera could be due to nonspecific reactivity (false positivity), due to antigenic cross-reactivity with HBV-like viruses or due to immunization to HBsAg disseminated in the environment^5^. Interestingly, antigen cross-reactivity has been observed between DCH and HBV^11^. Using a polyclonal serum specific for HBV core protein, the DCH antigen was identified in feline tissues by immunostaining. Accordingly, it is possible that the serological results reported in previous studies in dogs were actually due to cross-reactivity with HBV-like viruses, rather than to exposure of dogs to HBV.

With the exception of canine adenovirus type 1, the causative agent of infectious canine hepatitis (Rubarth's disease), few other viruses have been reported to target the liver of dogs^12^. Since hepadnaviruses are usually hepatotropic, it will be important to assess if DDH also has the potential to alter liver functionality and damage liver tissues in dogs. In our study, altered hepatic markers were found in DDH-positive dogs (Table 1). Also, we hypothesized that a potentially immunosuppressive condition of dogs, infection with *Leishmania* spp., could be associated with DDH, as observed for immunosuppressive retroviruses in humans and cats^13^. However, only 12/40 DDH-positive dogs (30%) had specific antibodies for *Leishmania*, versus 170/635 (26.7%) of the whole sera collection.

Finally, since hepadnaviruses in human and cats are able to cause long term infections^3,4^ and this can amplify the opportunity for virus transmission via blood and body fluids, it will be important to include DDH in the diagnostic screening of canine blood for dogs, to minimize potential risks, if any, for canine health.

## Methods

A total of 635 canine serum samples were collected from two different veterinary diagnostic laboratories located in Apulia region, Italy. The samples were originally collected from the veterinarian practitioners mainly for clinical examination and pre-operation testing. The study was approved by the Ethics Committee of the Department of Veterinary Medicine, University of Bari (authorization 04/2021). All experiments were performed in accordance with relevant guidelines and regulations.

Total DNA was extracted from the collected serum samples using the QIAmp cador Pathogen Mini Kit (Qiagen S.r.l., Milan, Italy). Canine sera were screened using a consensus PCR with pan-hepadnavirus primers targeting the polymerase ORF^14^. PCR products with sufficient DNA concentrations (>10 ng/μl) were directly sequenced by Eurofins Genomics GmbH (Ebersberg, Germany). Since initial sequencing revealed that the PCR-positive samples were highly similar (98–99% at nt level) to DCH, we commenced screening the canine samples with a DCH-specific qPCR, designed based on the DCH sequence of the polymerase region^13^, in parallel.

In order to acquire the complete viral genome sequence, positive samples were selected based on the genome copy number (DNA > 10^3^ copies/10 mL). For two of the selected samples, DNA enrichment was performed by multiply primed rolling circle amplification (RCA) technique^15^. Briefly, completion and circularization of relaxed viral DNA was obtained performing a completion/ligation (C/L) reaction using T4 DNA ligase (New England Biolabs, Ipswich, MA, USA) and T4 DNA polymerase (New England Biolabs, Ipswich, MA, USA), and the product of the C/L reaction was subsequently subjected to RCA using TempliPhi 100 amplification kit (GE Healthcare), as described previously^16^.

Twenty dog sera positive for DCH DNA were further tested by WB analysis to detect IgM and IgG antibodies anti-DCH core (DCHcAbs) by using the recombinant core protein of the feline strain ITA/2018/165-83^13^, expressed in baculovirus system (Fruci P., *Unpublished*). Each canine serum was diluted 1:50 and added with either horseradish peroxidase-conjugated goat anti-dog IgM (Bio-Rad, Italy) or with goat anti-dog IgG (Sigma-Aldrich, Milan, Italy), respectively at dilution of 1:1,000 and 1:3000.

A primer-walking strategy was used to generate the full-length DDH sequence, designing primers based on the DCH sequences available on GenBank. PCR was performed with Platinum II Hot Start DNA polymerase (Invitrogen). Sixty three complete genome sequences of hepadnaviruses were retrieved from GenBank. Sequence analysis was performed using Geneious software version 10.2.6 (Biomatters Ltd., Auckland, New Zealand). Alignment of the sequences was conducted using ClustalW plugin of the Geneious software. Phylogenetic analysis was conducted with MEGA-X version 10.0.5 software^17^ using the maximum likelihood method, the Hasegawa-Kishino-Yano model with a gamma distribution and invariant sites, and bootstrapping up to 1000 replicates.

## Supplementary Information

Supplementary Information.
