# Supplementary material for: A novel hepadnavirus in domestic dogs
Source: Sci Rep. 2022 Feb 21;12:2864. doi: 10.1038/s41598-022-06842-z (PMC8860997; doi:10.1038/s41598-022-06842-z)
Supplement: Supplementary file 1 — Supplementary Information. [file 41598_2022_6842_MOESM1_ESM.pdf]

Supplementary Table 1: Sample data used for this study. In bold the altered values of the hepatic markers.

Sample ID	Sex	Age in months	ALT 14-64 UI/L	AST 12-54 UI/L	ALP 20-120 UI/L	GGT 2-7.0 UI/L	Bilirubin 0.08-0.30 mg/dl	Nº of DDH DNA copies/mL
455-10	M	10	45	26	26	-	0.18	6,88 $x10^2$
570-31	M	24	58	37	71	-	0.13	4,03$x10^4$
570	F	12	52	24	40	2.2	0.18	2,84 $x10^4$
43-16	M	84	49	44	**331**	**10.4**	0.10	1,66 $x10^2$
43-18	M	108	52	47	66	4.4	0.18	3,60 $x10^2$
43	F	60	26	44	**223**	**1.1**	0.10	8,29 $x10^3$
232-5	M	60	17	16	49	3.2	0.13	1,36 $x10^2$
356-25	M	96	19	**72**	86	3.4	0.20	7,56 $x10^2$
356-27	M	132	50	29	**250**	3.5	0.13	5,79 $x10^2$
477-1	M	120	**110.5**	**152**	**855**	5	0.26	3,88 $x10^2$
477-4	M	144	62.6	**611**	31	1.9	0.23	9,33 $x10^2$
477-5	F	132	**79.7**	42	**1222**	6.4	0.14	2,64 $x10^2$
477-8	M	48	32	41	**167**	1	0.11	1,60 $x10^3$
477-10	M	108	**142.1**	31	**1480**	4.4	-	1,76 $x10^2$
477-30	F	240	**157.9**	52	**611**	**7.5**	0.19	1,77 $x10^2$
477-34	F	36	**0**	**639**	**1525**	**15.4**	**0.82**	6,89 $x10^3$
112-12	M	144	**274.2**	28	79	6.2	0.14	7,71 $x10^2$
112-20	M	120	54.7	33	**135**	4.9	0.28	1,47 $x10^3$
112-36	F	48	**163.5**	**107**	**1967**	**11.4**	**16.49**	6,88 $x10^2$

112-37	M	132	**307.5**	**170**	**1486**	**40.2**	**2.72**	7,66 $x10^2$
112-38	M	168	**86.5**	28	**132**	0	0.11	1,28 $x10^3$
112-39	M	24	19	44	**336**	0	0.17	6,02 $x10^2$
196-38	M	96	30.7	33	45	**1.6**	0.03	1,37 $x10^2$

Abbreviations: ALT, alanine transaminase; AST, aspartate transaminase; ALP, alkaline phosphatase; GGT, gamma glutamyl trasnpeptidase.
